# Genetics of Resistance and Pathogenicity in the Maize/*Setosphaeria turcica* Pathosystem and Implications for Breeding

**DOI:** 10.3389/fpls.2017.01490

**Published:** 2017-08-29

**Authors:** Ana L. Galiano-Carneiro, Thomas Miedaner

**Affiliations:** ^1^State Plant Breeding Institute, University of Hohenheim Stuttgart, Germany; ^2^Kleinwanzlebener Saatzucht (KWS) SAAT SE Einbeck, Germany

**Keywords:** *Exserohilum turcicum*, genomic selection (GS), *Ht* genes, marker-assisted selection (MAS), northern corn leaf blight (NCLB), recurrent selection (RS), resistance breeding

## Abstract

Northern corn leaf blight (NCLB), the most devastating leaf pathogen in maize (*Zea mays* L.), is caused by the heterothallic ascomycete *Setosphaeria turcica*. The pathogen population shows an extremely high genetic diversity in tropical and subtropical regions. Varietal resistance is the most efficient technique to control NCLB. Host resistance can be qualitative based on race-specific *Ht* genes or quantitative controlled by many genes with small effects. Quantitative resistance is moderately to highly effective and should be more durable combatting all races of the pathogen. Quantitative resistance must, however, be analyzed in many environments (= location × year combinations) to select stable resistances. In the tropical and subtropical environments, quantitative resistance is the preferred option to manage NCLB epidemics. Resistance level can be increased in practical breeding programs by several recurrent selection cycles based on disease severity rating and/or by genomic selection. This review aims to address two important aspects of the NCLB pathosystem: the genetics of the fungus *S. turcica* and the modes of inheritance of the host plant maize, including successful breeding strategies regarding NCLB resistance. Both drivers of this pathosystem, pathogen, and host, must be taken into account to result in more durable resistance.

## Introduction

*Setosphaeria turcica* (Luttrell) Leonard and Suggs (syn. *Helminthosporium turcicum*, teleomorph *Exserohilum turcicum* [Pass.] Leonard and Suggs), subclass Loculoascomycetidae, order Pleosporales, is a heterothallic ascomycete overwintering on host plant debris as dormant mycelium or as chlamydospores in the soil (Leach et al., 1977). Primary infections result from airborne conidia produced on maize debris which are transported by wind, rain, and seed borne inoculum (De Rossi and Reis, 2014). Infections are favored by temperatures between 15 and 25°C, dew periods of at least 4 h and 90–100% relative humidity (Levy and Cohen, 1983; Bentolila et al., 1991; Ogliari et al., 2005). The fungal mycelium penetrates directly the leaf cuticle and epidermis (Setyawan et al., 2016). Hyphae grow intracellularly into the mesophyll, proceed to vascular bundles, penetrate the xylem (Jennings and Ullstrup, 1957) and secrete HT (from *Helminthosporium turcicum*) toxin. HT toxin is composed of water soluble low molecular weight compounds inhibiting chlorophyll synthesis and are, therefore, phytotoxic (Bashan et al., 1995; Li et al., 2016). HT toxin is an important factor affecting pathogenicity, the pathogen’s ability to infect a resistant host, and virulence, which is the possibility to overcome non-specific host resistance genes (Vanderplank, 1984; Wathaneeyawech et al., 2015b). Moreover, the toxin induces disease symptoms and is associated with fungal aggressiveness (Bashan and Levy, 1992), the quantitative ability of a fungus to cause infection in the host (Vanderplank, 1984; Becher et al., 2013). This qualitative interaction between the resistance (*R*) gene of the host, and the Avirulence (*Avr*) gene of the pathogen directly affects conidial germination and ramification, and increases lesion size when the phytotoxin concentration is >250 ppm (Bashan et al., 1996). Hence, HT toxin is non-host specific (Yoka and Albertini, 1975; Petitprez et al., 1984; Bashan et al., 1995) and can affect many host plants (Mitchell, 1984).

**FIGURE 1:**
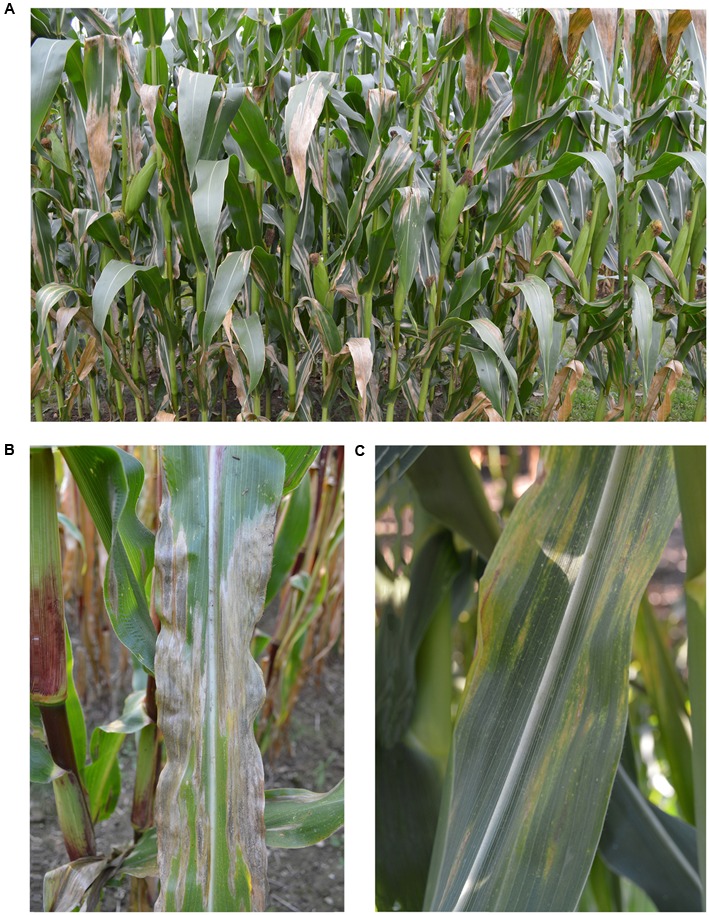
Northern corn leaf blight symptoms. **(A)** Symptoms of *Setosphaeria turcica* on a maize field with a susceptible cultivar. **(B)** Single leaf of a susceptible cultivar with symptoms of *S. turcica*, and **(C)** Single leaf with resistance reaction (Photos: Dr. Lucia Ramos-Romero, University of Göttingen, Germany).

About 14 days after infection, depending on host, pathogen, and environment, the first symptoms appear and expand further to a 2–30 cm long elliptical lesion of gray-green color which turns tan brown parallel to leaf margins (Welz, 1998). When no host resistance is available and optimal infection conditions persist, these lesions can coalesce and the entire leaf becomes blighted (**Figure 1**; Mengesha, 2013).

In the field, maize lesions grow 1.6–3.9 times faster at night than at the day, thus a day length shorter than 12 h enhances lesion growth. This is one factor making NCLB so severe in tropical and subtropical regions (Leach et al., 1977). Highly aggressive *S. turcica* isolates, however, can compensate suboptimal weather conditions resulting in severe epidemics also in temperate zones (Welz, 1998). In dead leaf tissue, sporulation commences with cloudy sky and 12 h day length as well as an extended period of high humidity (>90%) and a minimum of 14 h of dew period, resulting in higher spore production (Leach et al., 1977; Welz, 1998). This secondary inoculum spreads to other maize leaves, thus continuing the infection cycle.

Yield losses caused by NCLB depend on (i) host growth stage when the infection occurs, (ii) disease severity governed by the epidemic situation (Perkins and Pedersen, 1987), (iii) leaf insertion, (iv) level of host plant resistance, and (v) pathogen aggressiveness. Generally, yield losses are highest, when infection occurs before silking (Fajemisin and Hooker, 1974; Raymundo and Hooker, 1981; Ding et al., 2015) and the cob leaf is damaged (Welz, 1998). The percentage of yield loss due to the reduction in photosynthesis of the injured leaves under NCLB infection was around 63, 43, and 17% for an early maturing susceptible hybrid, a hybrid with quantitative resistance and intermediate maturity, and a hybrid with quantitative and qualitative resistances combined and late maturing, respectively (Raymundo and Hooker, 1981; Levy and Pataky, 1992). Additionally, NCLB may cause a reduction of feeding value and increases pre-disposition of maize to stalk rot (Hooker et al., 1965; Fajemisin and Hooker, 1974). To reduce these negative effects, fungicides, biological control, improved management practices, and resistant cultivars can be used.

Some carboxamides (Iprodione), phenylpyrroles (Fludioxonil), and sulfur compounds (Thiram) are the most efficient fungicides against *S. turcica* mycelium growth, the latter two are used in maize seed treatment (Rossi et al., 2015). Wathaneeyawech et al. (2015a) found that spraying contact fungicides (Chlorothalonil, Mancozeb) or azoles (Difenoconazole) 7 days before inoculation was the best timing with Difenoconazole being the most effective fungicide. Robertson and Pecinovsky (2016) demonstrated that the application of fungicides at five-leaf stage and at visible silk stage results in reduction of 50% in NCLB severity compared to the non-treated control or to application in five-leaf stage only. However, application of fungicides in maize is costly and can represent a risk to the farmer and to the environment when not handled properly.

Some *Bacillus* and *Enterococcus* species reduce *S. turcica* growth effectively (Sartori et al., 2015) and can be used as biological control agents. Moreover, chaetoglobosin A and chaetoglobosin C, metabolites produced by *Chaetomium globosum* N°05 strain (Ascomycota), have been reported to prevent symptom development on detached maize leaves (Zhang et al., 2013). Further research is necessary to identify the effect of these agents under field conditions and optimize their efficiency, their stability, and to address security issues (Zhang et al., 2013).

Among the management practices, tillage is the most important. In the last decades, reduced tillage or even no-tillage systems were largely exploited by farmers to prevent soil from erosion and to save time and costs. Consequently, the plant debris remains on the soil and enable the viable propagules of many fungi including *S. turcica* to survive the period where no host plant is grown. Tillage practices, therefore, indirectly reduce NCLB incidence and severity in the following crop (Sumner et al., 1981). Given this complex situation, only an integrated management system with improved cultural practices (crop rotation, burial or removal of crop residues) and resistant cultivars as the most important components should effectively control NCLB and avoid significant economic damage (Welz, 1998).

Resistant cultivars are important to control NCLB since they do not present additional costs for the farmer, do not harm the environment and reduce costs of seed production. Varietal resistance occurs in two forms in this pathosystem: (i) qualitative resistance governed by single, race-specific genes called *Ht* genes, and (ii) quantitative resistance, controlled by several to many genes each of which has only a small impact on disease resistance. In commercial cultivars both forms of resistance can be present.

Epidemiological aspects and management practices have been recently reviewed in detail by Hooda et al. (2017). This review, therefore, concentrates on population genetics of the fungus and resistance of the host including consequences for breeding. A high genetic variation in pathogenicity is indicative for a high evolutionary potential of a pathogen providing the basis for adaptation to fungicides and single resistance genes (McDonald and Linde, 2002). This often leads to low durability of resistances and, therefore, both drivers of this pathosystem must be analyzed to result in a sustainable management of resistance.

## Genetic Variation of S*etosphaeria turcica* Populations

*Setosphaeria turcica* populations are distinct among continents. In Mexico, the highest molecular diversity was found compared to *S. turcica* samples from Kenya, southern and northern China, Germany, Switzerland, France, and Austria. Mexico, therefore, is most likely the center of origin (Borchardt et al., 1998a). Tropical populations from Kenya, Mexico, and southern China are, in general, more genotypically diverse, have a lower gametic phase disequilibrium and a more even distribution of mating types when compared to temperate populations from Europe and northern China (Borchardt et al., 1998a). In addition, in the tropics, no clonal lineages were identified while in Europe, one third of the isolates had the same haplotype (Borchardt et al., 1998b).

Natural occurrence of the sexual stage, *Exserohilum turcicum*, was first reported in Thailand in 2013 (Bunkoed et al., 2014). Sexual hybridization enhances pathogen virulence by combining diverse virulences and generating new races (Bunkoed et al., 2014), thus playing a key role for pathogenic variation. The mating type is controlled by a single locus with two alleles (MAT-1 and MAT-2; Nelson, 1959). In tropical environments, an equal proportion of MAT-1 and MAT-2 was observed suggesting a frequent sexual hybridization that leads to a higher adaptation potential compared to temperate areas (Borchardt et al., 1998a). The reason why sexual hybridization occurs mainly in the tropics is still unknown.

Ferguson and Carson (2004) evaluated the diversity of *S. turcica* in the United States. by analyzing 251 maize isolates collected in the fields of 19 Eastern United States. A high pathogenic diversity was observed indicating the existence of sexual reproduction and a long-distance migration between states (Ferguson and Carson, 2004). The presence of nearly equal proportions of MAT-1 and MAT-2 alleles in some and dominance of MAT-1 or MAT-2 in other United States indicates the presence of both sexually and asexually reproducing populations depending on the region. Sexual reproduction tends to occur in the Southern United States, where the average annual temperature is higher, rather than in the Corn Belt (Ferguson and Carson, 2004).

Since *S. turcica* populations behave in large parts panmictic, it is impossible to identify diagnostic markers of virulence since recombination rapidly breaks down associations between the markers and the genomic region of interest. These markers would work, therefore, only with strictly asexual multiplication (Welz, 1998) or when directly placed within the avirulence gene.

The potential of a pathogen to adapt to quantitative disease resistances should be proportional to the level of genetic variation present in the fungal population (Fisher, 1930). According to McDonald and Linde (2002) pathogens with a mixed, i.e., sexual and asexual, reproductive system, high potential of genetic flow, and large population sizes are more likely to overcome host resistance and are, therefore, considered as “high-risk” pathogens. All these evolution forces apply for tropical *S. turcica* populations (Bergquist and Masias, 1974; Thakur et al., 1989; Borchardt et al., 1998a) resulting in highly diverse populations with a high probability of adapting to single-site fungicides or monogenic *Ht* genes.

## Qualitative Resistance to NCLB

The first element of plant defense against pathogens is based on PRR. PRR monitors the extracellular presence of PAMPs or DAMPs. When PAMPs or DAMPs are recognized by PRR, a signaling cascade response starts (Hurni et al., 2015). The pathogen has specific effectors that are injected into the host cytoplasm and suppress this plant response. When host proteins from the NBS-LRR family, like those coded by the *Ht* genes, recognize these effectors intracellularly, a second signaling cascade response starts (McHale et al., 2006; Dangl et al., 2013; Hurni et al., 2015) usually resulting in the death of the infected cell due to a hypersensitivity reaction. This reaction turns out to be qualitative and leads to “vertical” or race-specific resistance. The pathogens’ mutation from avirulence to virulence leads to a modification or suppression of these specific effectors. Consequently, the host plant cannot recognize the presence of the pathogen anymore leading to infection and subsequent pathogen reproduction. Due to its selective advantage the fungal subpopulation containing the virulence mutation can rapidly increase. When an *Ht* gene is not effective anymore due to a high frequency of the virulence mutation the resistance is colloquially called “broken” (McDonald and Linde, 2002), but indeed it is only ineffective due to a change in the pathogen population.

In the presence of qualitative *Ht* genes, the leaf presents chlorotic lesions with different levels of necrosis, wilting does not occur and sporulation is greatly reduced or even prohibited (**Figure 1**; Hilu and Hooker, 1963). The pathogen races are designated according to their virulence to the corresponding *Ht* gene (**Table 1**). Race 0 can infect only susceptible cultivars (*Ht0*) showing an incompatible host-pathogen interaction with all cultivars possessing an *Ht* gene. In contrast, race 1 is able to infect cultivars with *Ht1* gene due to a mutated avirulence (*Avr*) gene that turns the reaction into virulence. A single gene in *S. turcica* conditions the inheritance of virulence to *Ht1* gene and a gene-for-gene interaction occurs between the respective *Avr* gene and *Ht1* (Flor, 1956). The race with the highest virulence complexity known yet, race 123N, can infect all cultivars with the corresponding *Ht* genes. The expression of virulence to *Ht* genes depends on light and temperature conditions (Welz, 1998). Sixteen races of the pathogen could be, theoretically, identified by four *Ht* genes. Among them, 13 races have already been detected in northern China (Dong et al., 2008; Hooda et al., 2017) indicating a high race diversity of *S. turcica*.

**Table 1:** Gene-by-gene interaction between the pathogen and host plant (Welz, 1998).

Pathogen races	*Ht* gene reaction				
	*Ht0*	*Ht1*	*Ht2*	*Ht3*	*HtN*
0	+	–	–	–	–
1	+	+	–	–	–
2	+	–	+	–	–
3	+	–	–	+	–
N	+	–	–	–	+
12	+	+	+	–	–
2N	+	–	+	–	+
23	+	–	+	+	–
23N	+	–	+	+	+
123N	+	+	+	+	+

*- Incompatible reaction between Avirulence (*Avr)* gene and *Ht* gene, infection do not occur (= host resistance).*

*+ Compatible reaction between the *Avr* and *Ht* genes (= host susceptibility).*

Worldwide, race 0 showed the highest abundancy with a frequency of 55% (Welz, 1998). In Europe, race 0 represented 88% of the pathogen population while races N and 23N represented about 14 and 7%, respectively, in the 1990s (Welz, 1998). A monitoring from 2014 showed that in Central Europe, on average, race 0 occurred with 50.2% frequency among 255 isolates, 23.1% of them were identified as race 1 and 11% as race 3, and the races 3N, 123, 23, 2, 13, 23N, and 12 occurred, together, with a frequency of 15.7% (Hanekamp et al., 2014). There were, however, large regional differences. In the warmer areas of Central Europe, where maize growing is more abundant, race 0 represented only 25% of the described isolates and the remaining races were mainly virulent against *Ht1* and *Ht3* (Hanekamp et al., 2014).

Also, in the Eastern states of the United States race 0 declined from 83% in 1974 to 50% in 1990s most likely because of the use of *Ht1* gene in commercial maize hybrids as reported in a study with 242 isolates (Ferguson and Carson, 2007). Races 23 and 23N were only present in low levels. Accordingly, in the United States Corn Belt race 1 is nowadays more frequent than race 0 (Perkins, 2005; Pataky and Ledencan, 2006). Nine *Ht* genes have already been described in more detail (**Table 2**).

**Table 2:** Origin of qualitative resistance genes against *Setosphaeria turcica* and its defense reactions.

Genes	Location (bin)	Origin	Defense reaction	Reference
*Ht1*	2.08	Breeding material from the United States, Australia, Peru	Chloroses	Hooker, 1963, 1977; Ullstrup, 1963; Bentolila et al., 1991
*Ht2*	8.06	Breeding material from Australia	Chloroses	Hooker, 1977; Zaitlin et al., 1992
*Ht3*	7.04	*Tripsacum floridanum*	Chloroses	Hooker, 1981
*ht4*	1^a^	Breeding material from the United States	Chlorotic ring (ca. 1 cm)	Carson, 1995a
*HtM*	NA^b^	Variety from Puerto Rico	Full resistance	Robbins and Warren, 1993
*HtP*	2.08	Breeding material from Brazil	Full resistance or chloroses	Ogliari et al., 2005
*HtNB*	8.07	Landrace from Indonesia	Fewer lesions	Wang et al., 2012
*Htn1*	8.05	Landrace from Mexico	Fewer and delayed lesions	Gevers, 1975; Simcox and Bennetzen, 1993
*rt*	3.06	Breeding material from Brazil	Full resistance or chloroses	Ogliari et al., 2005

*^a^Short arm of chromosome 1 near the centromere.*

*^b^Not applied.*

In genotypes possessing the *Ht1* gene, sporulation is greatly suppressed in chlorotic lesions (Hilu and Hooker, 1963, 1964; Welz and Geiger, 2000) and lesion expansion is reduced since the hyphae spread only slowly from the xylem to the mesophyll of necrotic cells (Welz, 1998). This gene is partially dominant (Hooker, 1963; Dunn and Namm, 1970) and the degree of resistance depends on the genetic background where it occurs (Hooker, 1963; Calub et al., 1973; Leath and Pedersen, 1986). *Ht1* has been mapped on the long arm of chromosome 2 on bin 2.08, close to the RFLP markers *sgcr506* (Gupta et al., 1989; Welz, 1998) and *umc150B* (Bentolila et al., 1991; Welz, 1998).

*Ht2* presents similar chlorotic lesions but less necrosis than genotypes with *Ht1* (Welz, 1998). It is partially dominant (Hooker, 1977; Ceballos and Gracen, 1989). The gene *Ht2* has been mapped on the long arm of chromosome 8 in the *umc48*-*umc89* interval (Zaitlin et al., 1992; Welz and Geiger, 2000) on bin 8.06 (Zaitlin et al., 1992; Ding et al., 2015). A single dominant suppressor gene of *Ht2* was found in lines related to inbred ‘B14’ hampering backcross programs aiming to transfer *Ht2* gene (Ceballos and Gracen, 1989) into elite germplasm.

The gene *Ht3* was introgressed from *Tripsacum floridanum* into maize (Van Inghelandt et al., 2012) and it was mapped on bin 7.04 (Zhang et al., 2014). Another gene that confers race-specific resistance is the recessive gene *ht4* located on the short arm of the chromosome 1 near the centromere. In the presence of this gene the plant presents circular chlorotic halos of about 1 cm diameter (Carson, 1995a; Wang et al., 2012). Gene *HtM* was identified in inbred line ‘H102’ from the cross ‘C123’ × ‘PI 209135’ (‘Mayorbela’) (Robbins and Warren, 1993; Welz and Geiger, 2000). *HtP* was mapped on the long arm of chromosome 2 on bin 2.08 (Ogliari et al., 2005), *HtNB* gene, located on bin 8.07 was identified in the Indonesian line Bramadi and confers non-lesion resistance to *S. turcica* (Wang et al., 2012).

Gene *Htn1*, located on bin 8.05, tracing back to the Mexican landrace Pepitilla, confers partial resistance to NCLB (Welz and Geiger, 2000; Hurni et al., 2015). Differently from the genes *Ht1, Ht2*, and *Ht3, Htn1* delays lesion development up to 4 weeks after infection, reduces the number of lesions and delays the sporulation (Raymundo et al., 1981; Welz and Geiger, 2000). *Htn1* is effective to most NCLB races (Welz and Geiger, 2000), however, its level of resistance depends on environment and genetic background (Thakur et al., 1989). This gene has been mapped on the long arm of chromosome 8 (bin 8.05), while *Ht2* was mapped on bin 8.06 (Zaitlin et al., 1992; Simcox and Bennetzen, 1993; Ding et al., 2015). Hurni et al. (2015) associated a wall-associated receptor-like kinase (WAKs) with the *Htn1*. The WAKs attach the cell wall to the plasma membrane allowing these proteins to notify changes on cell wall structure (Brutus et al., 2010; Kohorn and Kohorn, 2012; Hurni et al., 2015). WAKs confer a new recognition pattern of the host defense immunity system since they can serve as DAMP receptors that recognize changes on cell wall during pathogen penetration in leaf tissue (Hurni et al., 2015). The recessive gene *rt* was identified by Ogliari et al. (2005) in the elite Brazilian line L40 and mapped on bin 3.06 (Ding et al., 2015).

Qualitative resistance usually leads to a high level of resistance when avirulent races dominate the fungal population. On the other hand, some *Ht* genes can quickly get ineffective in case of the occurrence of a virulent strain. Therefore, their use in breeding programs should be accompanied by regular analyses of race abundancy to select those genes that are still effective in the target region. In temperate environments, where the disease pressure is not as high as in the tropics, breeders readily introgress *Ht* genes since it is a faster strategy than improving NCLB resistance by means of quantitative resistance. Durability is hoped to be prolonged by pyramiding several *Ht* genes in the same cultivars. In tropical environments with high disease severity, high pathogen abundancy, and highly diverse *S. turcica* populations, *Ht* genes, however, provide only partial resistance (Welz and Geiger, 2000; Hakiza et al., 2004). The environment, mainly temperature and light intensity, may modify the expression of *Ht* genes and/or the corresponding avirulence genes in *S. turcica.* Maize breeders working in those regions are more reluctant to exploit monogenic resistances due to the higher risk of major gene resistance breakdown (Welz and Geiger, 2000; McDonald and Linde, 2002).

## Quantitative Resistance to NCLB

In environments, where the disease pressure and the genetic diversity of *S. turcica* populations are high, broad-based quantitative resistance to NCLB is essential. Maize cultivars with quantitative, “horizontal” or non-race specific resistance show a significant reduction of disease severity, but may still produce conidiophores and conidia (Hilu and Hooker, 1964). Typically, fewer and smaller lesions and a prolonged incubation period are observed in resistant hosts when compared to susceptible hosts (Ullstrup, 1970; Brewster et al., 1992; Smith and Kinsey, 1993; Carson, 1995b; Welz and Geiger, 2000).

Quantitative NCLB resistance is governed by many genes (polygenic). Most of the QTL have minor (0.5–5%) and only a few have major phenotypic effects (>20%). Entry-mean heritability (*h*^2^) of resistance is usually moderate to high: 0.53–0.95 as shown in a review by Welz (1998). Gene action varies with plant age, being purely additive in juvenile plants (Carson, 1995b) and dominance becomes gradually more important over the course of an epidemic (Schechert et al., 1997). Maternal and cytoplasmic effects are not important in this pathosystem (Geiger and Heun, 1989; Welz and Geiger, 2000), which differs from SCLB caused by *Cochliobolus heterostrophus* (Drechs.) Drechs. [anamorph: *Bipolaris maydis* (Nisikado and Miyake) Shoemaker]. Here, genotypes with CMS induced by the T cytoplasm are highly susceptible (Levings and Siedow, 1992).

Schechert et al. (1997) estimated genetic parameters for incubation period and AUDPC. These are important trait components for quantitative NCLB resistance being tightly correlated (*r* = ∼0.8) and highly heritable (*h*^2^= ∼0.8) (Welz and Geiger, 2000). The incubation period revealed mainly additive effects in crosses of susceptible by resistant lines while dominance effects were observed only in some crosses of resistant by resistant lines. For both resistance parameters, epistatic gene effects were not important (Schechert et al., 1997).

Quantitative trait loci for resistance were found on all chromosomes (Welz et al., 1999b; Wang et al., 2012; Chen et al., 2016) (**Table 3**). In the meanwhile, multiple-resistant loci including NCLB resistance loci, have been detected. McMullen and Simcox (1995), Wisser et al. (2006), and Jamann et al. (2014) identified clusters of multiple disease resistance factors in bins 3.04, 6.01, and 1.06, respectively. Wisser et al. (2006) revealed strong evidences of association between resistance loci for NCLB, head smut, and common rust resistance. In a fine mapping study, a QTL was found on chromosome 1 conferring resistance to NCLB, Stewart’s wilt (caused by *Pantoea stewartii*) and common rust (caused by *Puccinia sorghi*) (Jamann et al., 2016).

**Table 3:** Synthesis of some QTL mapping studies using composite interval mapping (CIM).

Parents		Test site^a^	Population size	% of phenotypic variance		Reference
Resistant	Susceptible			Range^b^	Total	
**Incubation period**				
Mo17^c^	B52	Tr	121	9.8–38.0	40.9	Dingerdissen et al., 1996
CML202	Lo951	Tr	194	7.0–11.8	52.2	Schechert et al., 1999
B73	Mo17	Te	302	4.1–6.9	51.6	Balint-Kurti et al., 2010
DK888	S11	Te	96	NA^d^	61.0	Chung et al., 2011
**Disease severity**				
Mo17	B52	Te	150	7.5–13.4	51.5	Freymark et al., 1994
D32; D145^c^		Te	220	5.2–20.9	61.5	Welz et al., 1999b
CML202	Lo951	Tr	194	7.2–24.8	55.4	Welz et al., 1999a
IL731a; W6786		Te	157	4.6–10.7	49.4	Brown et al., 2001
K22	By815	Te	207	6.7–15.5	56.3	Chen et al., 2016
**AUDPC**				
Mo17	B52	Tr	121	9.8–18.3	47.8	Dingerdissen et al., 1996
CML202	Lo951	Tr	194	6.9–18.3	55.8	Schechert et al., 1999
CML52	B73	Te	98	NA^d^	12.0	Chung et al., 2011
Historical minnesota inbreds		Te	284	NA^d^	55.0	Schaefer and Bernardo, 2013

*^a^Tr: tropical, countries below the equator, all locations in Kenya were assumed to be tropical. Te: temperate.*

*^b^Phenotypic variance explained by the smallest and largest QTL effect, respectively.*

*^c^Moderate resistance. ^d^Not given.*

*Three traits related to NCLB resistance are shown: incubation period, disease severity (affected leaf area or lesion width), and AUDPC.*

Van Inghelandt et al. (2012) demonstrated that 15.95% of genotypic variance was explained by four QTL on chromosome bins 2.08, 5.03, 6.05, and 7.02 in a GWAS of 1487 inbred lines. A SNP marker on bin 5.03 was identified in a region of unknown function, while a SNP with minor effect was located in the *GPC4* gene (bin 5.05), involved in sugar metabolism and showing expression differences upon anaerobiosis as well as heat shock (Russell and Sachs, 1992; Van Inghelandt et al., 2012). The SNP on bin 7.02 is located in a *DBF1* gene, which is a member of the Apetala 2/Ethylene transcription factor family (Kizis and Pagès, 2002; Van Inghelandt et al., 2012) and has a role in abiotic stress responses. Plants that are sensitive to drought stress have a tendency to show early senescence symptoms. Since *S. turcica* is a necrotrophic pathogen, NCLB tends to progress quicker in senescing tissue (Rupe et al., 1982; Van Inghelandt et al., 2012), mainly after anthesis (Rupe et al., 1982).

Another GWAS study was conducted by Ding et al. (2015) where 999 inbred lines were analyzed using 56,110 SNPs. They significantly associated 12, 14, and 19 markers to the traits AUDPC, mean disease rating, and final disease rating, respectively. Genes associated to two or three of the traits simultaneously were identified on chromosomes 4, 7, and 10 and the functional annotation of three of these genes correspond to biotic stress resistance, such as the SANT domain-associated protein and the DNA-binding gene WRKY.

## Potential Candidate Genes

Besides candidate genes derived from GWAS, other genes have been suggested earlier. DIMBOA (2,4-dihydroxy-7-methoxy-1,4-benzoxazin-3-one), an antimicrobial substance in maize, affects *S. turcica*, European Corn Borer, and *Fusarium* spp. Plants homozygous for the mutant gene *bx1* (benzoxazinless 1) do not produce DIMBOA and the host becomes extremely susceptible to NCLB. The genes *bx1* to *bx5* are located on the short arm of chromosome 4S (McMullen and Simcox, 1995). In another study, Frey et al. (1997) assigned a different bin position from *bx1* to *bx5* on chromosome 4. The hypothesis that variation at the *bx1* locus is responsible for DIMBOA production is less probable to be validated. An *in vitro* experiment confirmed the significant inhibition of *S. turcica* mycelium growth by DIMBOA (Rostás, 2007). With 0 μg ml^-1^ DIMBOA, the mean growth size of the mycelium was about 2 cm^2^, while with 250 and 750 μg ml^-1^ the mean size was 1.5 and 1.0 cm^2^, respectively (Rostás, 2007). Besides inhibition of mycelium growth, DIMBOA can also affect spore germination of *S. turcica* (Couture et al., 1971; Long et al., 1975).

Lesion-mimic mutant (*Les/les*) is one of the most common stress phenotypes in plants (Johal, 2007). Some of these lesion-mimic mutants can induce similar symptoms like NCLB (Hoisington et al., 1982). *Les1*, a lesion-mimic dominant mutant gene located on the short arm of chromosome 2 in maize, induces lesion formation with specific size, shape, and coloration (Hoisington et al., 1982). *Les 1*, therefore, could be involved in induction of NCLB necrosis. In total, more than 50 *Les/les* mutants have been identified in maize (Walbot et al., 1983; Johal et al., 1995; Buckner et al., 2000; Johal, 2007) and it is assumed that more than 200 *Les/les* mutants may exist (Walbot et al., 1983; Johal, 2007). Further research is necessary to explore this topic in relation to NCLB.

Micro RNAs (*miRNAs*) are gene expression regulators that are related to many stress responses. Wu et al. (2014) demonstrated that *miR811* and *miR829* confer a high degree of resistance to NCLB. The relationship between *S. turcica* and *miRNAs* remains to be explored (Wu et al., 2014).

## Implications for Breeding of NCLB Resistance

Successful resistance-breeding programs need effective resistance sources, testing systems to reliably assess genetic differences in resistance, and adequate selection and breeding methods.

Resistance sources can be identified especially in areas where the disease pressure is high. Eastern and Southern Africa, Latin America, China, and India are hot spots for the development of NCLB preferrently in the mid-altitude regions, 900–1600 m above the see level, where long dew periods, moderate temperatures, and short day length lead to a high disease pressure (Renfro and Ullstrup, 1976; CIMMYT, 1988; Welz and Geiger, 2000). Materials from Kenya (Muiru et al., 2007) and Uganda (Adipala et al., 1993), for example, have been demonstrated to be highly resistant to NCLB. More resistance sources and their origins are listed, for example, in Welz and Geiger (2000), Ding et al. (2015), and Hooda et al. (2017).

Northern corn leaf blight phenotypic evaluations are usually assessed in the field in adult-plant stage. Artificial inoculation ensures high NCLB pressure and uniform disease distribution in the nursery. This maximizes genetic differentiation and, thus, ensures high heritability and large potential selection gains (Welz, 1998). An advisable inoculation technique for large populations is to collect infected leaves, ideally 4–6 weeks after anthesis in order to avoid a mix of *S. turcica* and other leaf pathogens (Hooker, 1973). The leaf samples must be kept dry and cool to avoid the loss of *S. turcica* pathogenicity and the ability to sporulate. Crushed infected leaves are placed about 10 days before flowering in the maize whorl, ideally in the same field locations where the infected leaves were collected (Hooker, 1973; Hurni et al., 2015). The infection tends to be higher when the inoculum is added during or just after light rain or prior to irrigation (Hooker, 1973). When the weather is dry and hot, the secondary spread of inoculum may happen naturally, in unfavorable weather conditions a second spread of inoculum and/or sowing spreader rows of susceptible genotypes may be necessary (Hooker, 1973). Craven and Fourie (2016) visually assessed NCLB lesions in the field at the growth stages of visible silks (R1), kernels start to fill (R2), milk stage (R3), top part of kernel filled with starch (R4), and dent stage (R5), respectively (Anonymous, n.d.). Based on these multiple disease ratings the incubation period and AUDPC can be estimated (Welz and Geiger, 2000). In routine breeding programs, field evaluation is realized one to three times, depending on the development of disease symptoms. Scoring is based on disease severity (**Table 4**) in the field. Ratings are performed plotwise with scores ranging from 1 to 9 or 1 to 5 where the lowest number represents a plot without NCLB symptoms and the highest number is a plot with severest disease symptoms. NCLB symptoms can be confounded by other diseases such as Stewart’s wilt caused by *Pantoea stewartii* in locations where both diseases occur. A microscopic examination of leaf tissue can easily differentiate both disease symptoms (Pataky, 2004).

**Table 4:** Scoring method of NCLB incidence on the field useful for assessing large maize populations (Hurni et al., 2015).

Score	Phenotype
1	Plants do not show disease symptoms
2	First small lesions appear on few plants per row and occupies less than 5% of leaf surface
3	Many plants per row present in one leaf level lesions occupying 5–10% of the leaf
4	Many plants per row present in several leaf level lesions occupying 10–20% of the leaf
5	Lesions occupying 20–40% of the leaf and start to merge
6	Lesions occupying 40–60%
7	Lesions occupying 60–80%. Half of the leaf is dry due to disease infection
8	Lesions occupying 80–90%. More than half of the leaf is dry due to disease infection
9	Lesions occupying 90–100%. Nearly the whole plant is dry due to disease infection

Evaluation of NCLB resistance in line *per se* performance is tightly correlated (*r* = 0.94–0.98) to its GCA (Schechert et al., 1997). The high correlation for *per se* evaluation corroborates to the fact that gene expression of NCLB resistance is mainly additive (Abera et al., 2016). Maize resistance-breeding programs should allocate their resources in early selection stages, therefore, for *per se* evaluation of NCLB resistance rather than for testcross performance (Schechert et al., 1997). However, the disease shows some heterosis for resistance (18–27%) and consequently experimental hybrids should be also tested for NCLB resistance in a later selection stage in order to exploit this heterosis (Schechert et al., 1997).

Some studies reveal low (Balint-Kurti et al., 2010) to moderate correlations (Van Inghelandt et al., 2012; Bernardo and Thompson, 2016) between flowering date and NCLB severity with early flowering lines being more susceptible. However, none of the studies shows a clear correlation pattern between flowering date and disease development.

The choice of the most adequate resistance type in a breeding program depends on the population structure and the evolutionary capacity of a pathogen (McDonald and Linde, 2002). In environments where the pathogen population is highly diverse and the gene or genotype flow is high quantitative resistance or exploiting qualitative resistances by using multilines or cultivar mixture are recommended. Producing complex hybrids, such as three-way and double-cross hybrids, with inbred lines differing in resistance gene(s) can be another strategy to retard gene erosion (McDonald and Linde, 2002), since these complex hybrids are heterogeneous and, therefore, present a large genetic variation within the cultivar (Welz, 1998). They are routinely produced in some breeding programs due to the lower costs of hybrid seed production compared to single-cross hybrids. In environments, where the pathogen diversity is lower, the use of qualitative resistances is recommended since it is easier to identify diseased plants and can be employed in a breeding program more easily (McDonald and Linde, 2002).

While *Ht* genes can be easily introgressed by multiple backcrosses with or without molecular markers, improving quantitative resistances can be accomplished by RS procedures. The main objective is to improve the frequency of favorable alleles and maintain a sufficient genetic variation in order to increase the population performance in the subsequent cycles (Falconer and Mackay, 1996). This method includes the development of progenies from a population with some resistance level, evaluation of progenies and selection of the best progenies for recombination of selected individuals for the next selection cycle. The selection response to this breeding method depends, among others, on the square root of the heritability. In NCLB resistance tests, the heritability is usually moderate to high; therefore, it is expected to achieve rapid improvement progress by RS, considering a large genetic variance within the source population (Schipprack, personal communication). It is important, however, that the presence of effective *Ht* genes mask the selection of quantitative resistance and, thus, should be avoided when breeding for quantitative resistance (Welz, 1998).

RS has been successfully used for improving NCLB resistance by several groups. Ceballos et al. (1991) used RS for NCLB resistance improvement and observed with 19% per cycle a high selection gain. Carson (2006) studied the response to selection of two traits related to partial resistance to NCLB: latent period and lesion length. Selection gain per cycle for latent period was higher than for lesion length, 20–27% and 14–18%, respectively, after three cycles of RS with a selection intensity of 10% per cycle. Ayiga-Aluba et al. (2015) studied the efficiency for selection of NCLB traits through a S1 RS program across two cycles. Among other traits the measurement of AUDPC provided a reduction of 26% per cycle indicating that the S1 RS was efficient. Ribeiro et al. (2016) applied seven cycles of RS among 200 half-sib popcorn families and also concluded that selection was effective. Brewbaker (2009) released a synthetic population after 10 cycles of RS to NCLB resistance without giving disease scoring data. The selection was conducted in Hawaii in a location where the disease incidence was high and the known *Ht* genes were not effective anymore.

When the NCLB resistance level in a population is already high enough, a multi-stage selection integrated in the commercial breeding program can be routinely applied. With this method, selection is realized through successive screenings of different sets of traits per generation. In each screening step, different information and selection intensity are used for selection (Cunningham, 1975). NCLB resistance can be selected in early stages of inbred line development because heritability is high. Other qualitative and qualitative traits, including agronomic traits and other disease resistances, can be simultaneously selected.

Marker-assisted selection is an important breeding tool when selecting for resistant material, especially when introgressing *Ht* genes or major QTL via backcrossing. With molecular markers, it is possible to identify in the early stages of plant development plants containing the gene or QTL of interest (foreground selection), increase the proportion of recurrent parent genome (background selection), and reduce linkage drag (Miedaner, 2016). Codominant SSR markers linked to the known *Ht* genes *Ht1, Ht2*, and *Htn1* have already been identified, such as *bnlg1721* and *umc1042* being closely linked to the resistance gene *Ht1* (*R*^2^ = 0.2948 and 0.2626, respectively, *p* < 0.0001, Puttarach et al., 2016). These SSR markers can also be used to select for absence of *Ht* genes during selection for quantitative resistances, thus avoiding results biased by the presence of race-specific genes.

For using QTL, it is necessary to validate them prior to the backcross steps in independent populations or materials derived from the original crossing, like near-isogenic lines. Asea et al. (2009) validated a QTL on bin 3.06 while Chung et al. (2010) validated the QTLs qNLB1.02B73 and qNLB1.06Tx303, identified in bin 1.02 in genotype B73, and bin 1.06 in line Tx303, respectively. The identification of molecular markers closely linked to the gene or QTL of interest is also crucial for a successful MAS. Asea et al. (2012) demonstrated that the use of markers linked to the target QTL is highly efficient and a cost-effective tool to improve foliar disease resistances in maize. Some dominant SCAR markers such as *SCA07496, SCA16420, SCB09464*, and *SCE20429* were identified and can be successfully used to identify NCLB resistant genotypes (Khampila et al., 2008) although it is not possible to discriminate homozygous from heterozygous resistant plants. In maize, large SNP marker chips are available such as SNP50 Beadchip (Illumina, Inc.) containing 56,110 SNPs (Ganal et al., 2011) that have been used in quantitative resistance studies to NCLB (e.g., Schaefer and Bernardo, 2013; Ding et al., 2015; Chen et al., 2016).

Improving quantitative NCLB resistance by combining several QTL is nowadays considered as less effective (Bernardo, 2008; Xu and Crouch, 2008; Jannink et al., 2010). In MAS, firstly QTL are identified and later on estimates of their effects are computed. This leads to a long procedure and a biased estimation, especially when only QTL with small effects are detected (Lande and Thompson, 1990; Jannink et al., 2010). GS seems to be more promising than MAS since it enables the simultaneous estimation of all marker effects of a genotype and, thus, can be effectively used in selecting quantitative traits, even when only small-effect QTL are available (Jannink et al., 2010). Prerequisites for GS are (i) large training populations segregating for NCLB resistance that are intensively phenotyped across locations and years and genotyped by high-density markers, (ii) adequate GS models, and (iii) genotyped test populations that are selected by using the most appropriate GS model. Thus, the most resistant genotypes to NCLB are predicted on the basis of their GEBV (Jannink et al., 2010). Thus, greatly reduces the amount of necessary test units in the field because only the most resistant predicted progenies are field tested. Thus, resources can be reallocated in order to increase selection gain per breeding generation by testing larger populations. Technow et al. (2013) demonstrated a high prediction accuracy for NCLB resistance of 0.71 (dent gene pool) and 0.69 (flint gene pool) when using the GBLUP model, thus encouraging the application of GS.

## Conclusion

Northern corn leaf blight resistance can be monogenically or polygenically inherited. The most adequate resistance type used in a breeding program depends on the population structure and the evolutionary capacity of the pathogen. In environments with lower disease pressure and low diversity of *S. turcica* populations, like in the temperate regions, introgression of *Ht* genes by recurrent backcrossing might be favored, because it is easy to accomplish for the breeder. Durability, however, might also here be restricted. NCLB shows to be more severe in the subtropics and tropics compared to temperate environments due to the shorter day length, higher humidity, and likely higher frequency of sexual reproduction of the fungus. Here, quantitative resistance to NCLB should be the main focus of resistance-breeding programs. Population improvement should favorably be accomplished by RS or multi-stage selection. For introgressing major QTL, molecular markers could accelerate the process. GS procedures might help to effectively accumulate the described small-effect QTL in high yielding maize materials.

## Author Contributions

ALGC conceived and wrote the manuscript. TM drafted and edited the manuscript. Both authors approved the final version to be published.

## Conflict of Interest Statement

The project was financially supported by KWS SAAT SE, Einbeck, Germany. ALGC was also employed by KWS SAAT SE. The authors confirm that this did not affect the design of the study, or the analysis of the results. TM has no potential conflicts of interests to disclose.

## Abbreviations

AUDPC: area under the disease progress curve
BLUP: best linear unbiased predictor
CMS: cytoplasmic-male sterility
DAMP: danger-associated molecular patterns
ETI: effector-triggered immunity
GBLUP: genomic best linear unbiased predictor
GCA: general combining ability
GEBV: genomic estimated breeding value
GS: genomic selection
GWAS: genome wide association study
MAS: marker-assisted selection
NBS-LRR: nucleotide-binding site-leucine-rich repeat
NCLB: northern corn leaf blight
PAMP: pathogen associated molecular patterns
PRR: plasma membrane-anchored pattern recognition receptors
QTL: quantitative trait loci
RS: recurrent selection
SCAR: sequence characterized amplified region
SCLB: southern corn leaf blight
SNP: single nucleotide polymorphism
SSR: single sequence repeat
